# Analysis of mutual couplings in a concentric circular ring plasmonic optical antenna array

**DOI:** 10.1038/s41598-017-10690-7

**Published:** 2017-09-08

**Authors:** Guiru Gu, Lin Li, Yingjie Zhang, Thitikorn Kemsri, Xuejun Lu

**Affiliations:** 1 Department of Physics, Stonehill College, 320 Washington Street, Easton, MA 02357 USA; 2 Department of Electrical and Computer Engineering, University of Massachusetts Lowell, One University Avenue, Lowell, MA 01854 USA

## Abstract

In this paper, we report the analysis of a concentric circular ring plasmonic optical antenna (POA) array using a simple lumped coupled circuit (LCC) model. The currents in the circular rings of the POA array and their mutual couplings are analyzed using the LCC model. The results agree well with the numerical simulation using CST’s Microwave Studio®. The LCC model reveals the mutual couplings between the antenna rings. It is found that the mutual couplings are not only between the adjacent antenna rings, but also involve their second (2^nd^) nearest or farther neighbors. Since the near-fields of the optical antennas are related to the currents in the optical antennas, the LCC model provides a useful tool for the analysis of the near-field and their mutual interactions in the circular ring POA array.

## Introduction

Antennas are critical components in transmitting and receiving electromagnetic waves in the r.f., microwaves, and millimeter-wave spectral regimes. Optical antennas^1–11^ are their counterparts in the optical spectral regimes such as visible, near infrared (IR), and middle wave infrared (MWIR) and longwave infrared (LWIR). Optical antennas and their applications in controlling light sensing and emission properties have been extensively studied^1–5^. Depending on the optical antenna structures, localized surface plasmonic resonance (LSPR) modes^12, 13^ can be excited in optical antennas^1–5, 14–16^, which are referred to as plasmonic optical antennas (POAs). POA enhanced quantum dot infrared photodetectors (QDIPs) have been reported with enhanced photocurrents and directional antenna gains^6, 17, 18^. The analysis of the near-field of POAs and their roles in the plasmonic enhancement have also been reported^6^.

In a POA array with multiple antenna elements, the near-fields of the POA array can be significantly affected by the mutual couplings among the antenna elements. In addition, the requirement to satisfy the electromagnetic boundary conditions at multiple antenna elements also makes it complicated to analytically calculate their near-fields and the couplings through their near-fields^19^. Numerical simulation methods such as the finite-difference-time-domain (FDTD)^20^, the method of moments (MoM)^21^, the fast multipole method (FMM)^22^, and the integral equation solver^23^, have been developed to numerically calculate the near-field distributions. These numerical methods are very effective in numerically calculating the near-fields as well as performing the design validations and obtaining optimal designs. However, the underlying physics, such as the LSPR modes excitation, the near-fields and current distributions and their relationships with the interactions of the multiple antenna elements, are generally hard to unveil from the numerical data.

Since the near-fields can be uniquely defined by the currents in optical antennas due to the uniqueness theorem^24, 25^, it is possible to determine the near-fields from the currents in the antennas. Therefore, the near-fields and their couplings can be available once the current distributions and their couplings are obtained. Mutual couplings in r.f. antenna arrays have been extensively studied using equivalent circuit analysis^26^. Terahertz (THz) optical antennas have been modeled using the transmission line (TL) theory and the surface current can be calculated using the TL theory^27, 28^. However, to our best knowledge, there is no report on the analytical modeling of mutual couplings of POA arrays and their dependence on the POA array elements ﻿﻿using a circuit model﻿.

In this letter, we present a simple lumped coupled circuit (LCC) model to analyze a concentric circular ring POA array. The current distributions of the array elements and their mutual couplings are analyzed using the LCC model. The results are compared with the numerical simulation data using CST’s Microwave Studio®. The two methods show good agreement. The mutual couplings between the POA antenna elements are found to be not just between adjacent antenna elements, but also involve 2^nd^ nearest or farther antenna elements.

## Results and Discussion

**Figure 1:**
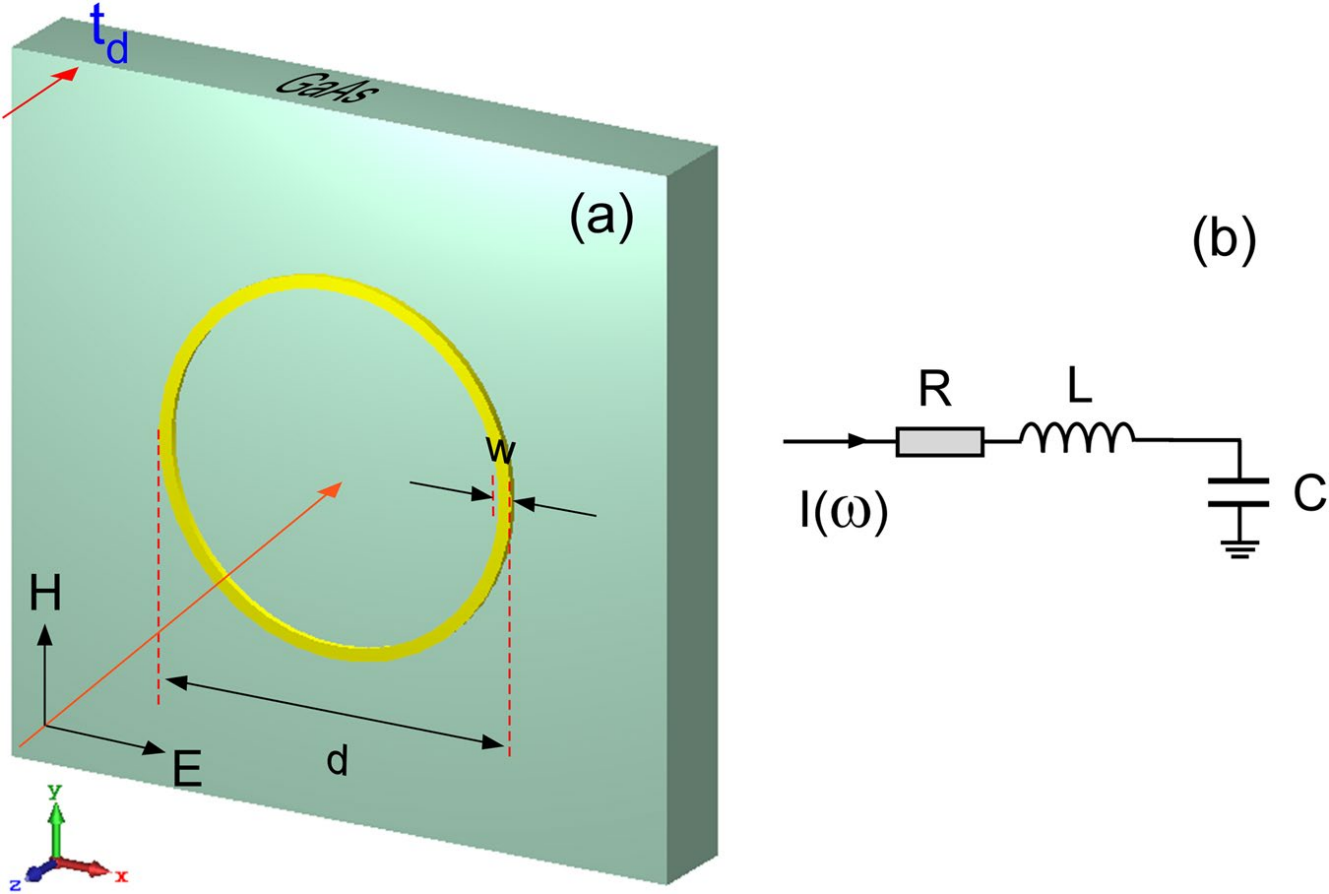
Schematics of a single metallic (gold) circular ring POA. (**a**) Physical layout; (**b**) equivalent series RLC circuit. The incident light is a surface normal plane wave traveling in the -z direction with the E-filed aligned in the x-direction and the H-field in the y-direction. The magnitude of the E-field is set to 1 V/m.

Before modeling of the multiple element concentric circular ring POA array, we first analyze a single circular ring POA and its equivalent lumped RLC circuit. Figure 1(a) shows the schematic layout of the circular ring POA. It is a metallic (gold) ring on a GaAs substrate. The thickness of the antenna ring *t*_*m*_ is 30 nanometers (nm), and the thickness of GaAs substrate is *t*_*d*_ = 0.35 µm. The outer diameter and the width of the metallic ring are labeled as *d*, and *w*, respectively. The incident light is a surface-normal plane wave propagating in the −z direction (i.e. top illumination).

Figure 1(b) illustrates the equivalent lumped circuit. It is a simple series RLC circuit. The current is *I(ω*) in the frequency domain.

The single POA was numerically simulated using CST’s Microwave Studio®. The incident light is a surface normal plane wave traveling in the -z direction (i.e. top illumination). The H-field is in the y-direction, and the E-field is in the x-direction with a magnitude of 1 V/m throughout the paper. Since the circular rings are symmetric, other linear polarizations give the same results. Open boundary conditions were used in all simulations. The incident light excites LSPR modes in the circular ring POA and generates surface current in the ring.

**Figure 2:**
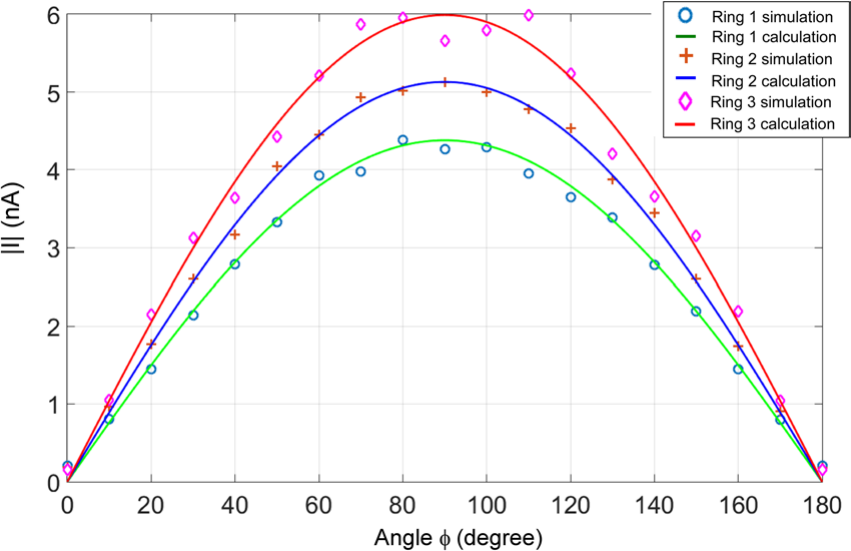
Current distributions in the individual rings at their corresponding resonant wavelengths. Dots, crosses, and diamonds: the current values from the CST simulation; Solid lines: calculated current using the |I| = |Imax|sin(ϕ) plots. The resonant current distributions follow the sinusoidal standing wave current distribution in the half-wave antennas.

The resonant condition of the fundamental LSPR mode can be written as:

1 $${k}_{sp}\frac{\pi d}{2}=\pi ,$$

where *k*_*sp*_ is the wave vector of the surface plasmonic wave. At the resonance, the LSPR mode induced surface current shows a sinusoidal-type distribution along the ring with a maximum current *I*_*m*_(ω) at the top (i.e. x = 0, and y = *d/2*) and bottom (i.e. x = 0, and y = −*d/2*) of the ring. Figure 2 shows the simulated current distributions (dots, crosses, and diamonds) in the three individual rings﻿, Ring 1, Ring 2, and Ring 3 with the outer diameters of 1.2 µm, 1.4 µm, and 1.6 µm, respectively. The currents are at their corresponding resonant frequencies of 30 THz, 26 THz and 23 THz, respectively. The widths of the rings and thickness of the substrate are kept at *w* = 0.05 µm and *t*_*d*_ = 0.35 µm, respectively. Under the plane wave incidence, the current distributions in the upper and lower parts of each ring are symmetric. Therefore, only upper half ring (i.e. angle 0 ≤ ϕ ≤ 180°) is analyzed. The other half is the same due to the symmetry.

The currents are also calculated using the sinusoid standing wave formula in a standard half-wave antenna, i.e. |I| = |Imax|sin(ϕ). The calculated sinusoidal current distributions (solid lines) are also plotted in Fig. 2. The current distributions follow the sinusoidal standing wave current distribution in a half-wave antenna.

**Figure 3:**
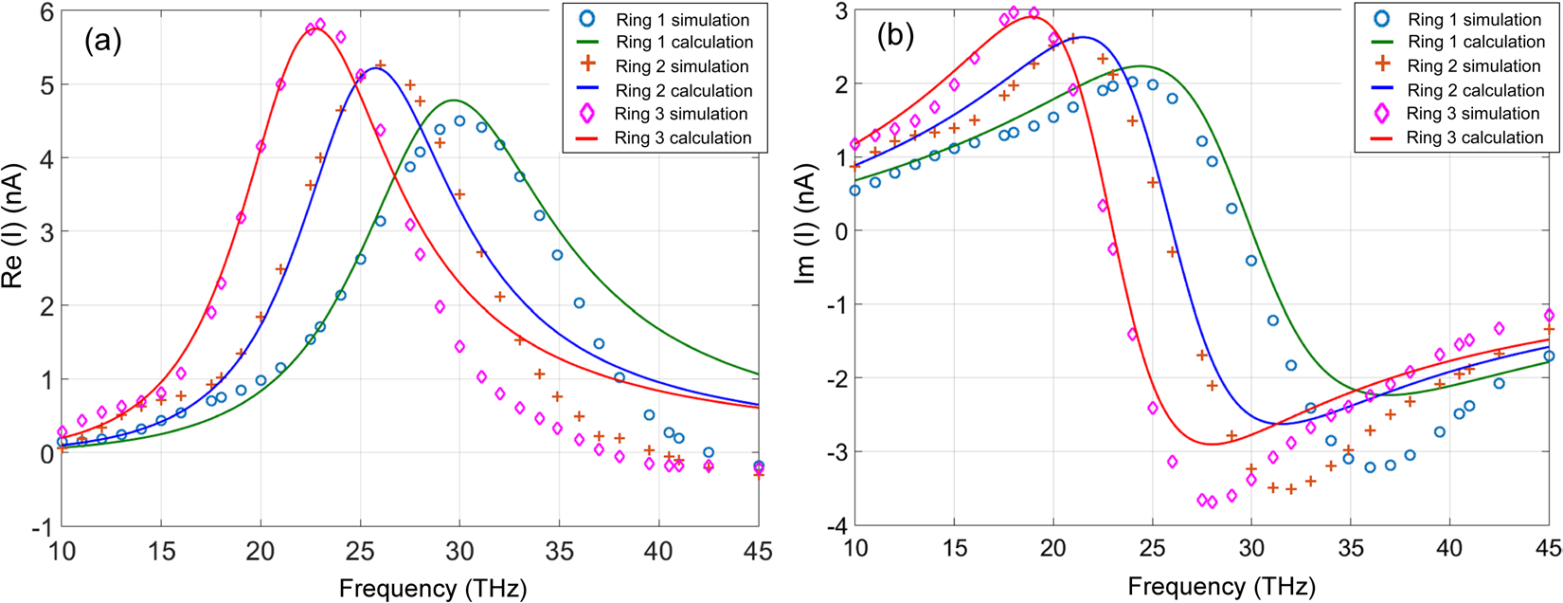
Surface currents in individual uncoupled circular rings with different diameters of 1.2 µm, 1.4 µm, and 1.6 µm for Ring 1, Ring 2, and Ring 3, respectively: (**a**) *Re* (*I*); (**b**) *Im*(*I*). The solid curves are calculation data using the lumped circuit models, and the points by the “o”, “+” and “◊” are numerical simulation data points using CST’s Microwave Studio®.

Figures 3(a) and 3(b) show the simulated real parts *Re*(*I*) and imaginary parts *Im*(*I*) of the surface currents *I*_*m*_(ω) of different circular rings (dots, crosses, and diamonds).

The surface current *I*(ω) in the equivalent lumped RLC circuit can be written as:

2 $$I(\omega )=\frac{{V}_{0}}{R+j\omega L+\frac{1}{j\omega C}},$$

where *ω* is the angular frequency of the incident light, R is the resistance, L is the inductance, C is the capacitance, and *V*_0_ is the induced voltage in the lumped circuit.

The resistances R are the Ohmic resistances of the individual rings:

3 $$R=\rho \frac{\pi ({R}_{in}+{R}_{out})/2}{\sqrt{2}W{t}_{m,sk}},$$

where *ρ* = 2.44 × 10^−8^ Ω-m is the resistivity of gold (Au)^29^, and *R*_*in*_ and *R*_*out*_ are the inner and outer radius of the ring, respectively. *t*_*m*,*sk*_ is the skin depth.

4 $${t}_{m,sk}=\sqrt{\frac{\rho }{\pi f\mu }},$$

where *f* is the frequency, and *µ* is the permeability of the metallic (gold) rings. Since the skin depth *t*_*m*,*sk*_ is frequency-dependent, the R also changes with the frequency. The $\sqrt{2}$ factor in Eq. (3) is due to the use of the I_max_ in the power loss calculation. The calculated Ohmic resistances are 47.2 Ω, 47.5 Ω, and 51.2 Ω for Ring 1, Ring 2, and Ring 3 at their resonant frequencies of 30 THz, 26 THz and 23 THz, respectively.

The voltages (V_0_) of the rings are calculated from the simulated E-field distribution along the rings, i.e.:

5 $${V}_{0}={\int }_{0}^{180}\vec{E}\cdot \hat{\varphi }Rd\varphi ,$$

The calculated voltage values are 2.0 × 10^−7^ V, 2.3 × 10^−7^ V, and 2.9 × 10^−7^ V, for Ring 1, Ring 2, and Ring 3, respectively.

The inductance of the circular rings with the rectangular cross section can be calculated using Eq. (6) given by Frederick W. Grover^30^:

6 $${L}_{u}=0.002{\pi }^{2}(\frac{2R}{{t}_{m}})RK^{\prime} ,$$

where L_u_ is the inductance in microhenries (µH), R and t_m_ are in centimeters (cm), and K’ is the is a dimensionless factor that depends upon the (2 R/t_m_) ratio and the radius of the ring antennas. K’ values are given by Frederick W. Grover^30^. The inductance L of the half ring is thus:

7 $$L=0.002{\pi }^{2}(\frac{2R}{{t}_{m}})\frac{RK^{\prime} }{2},$$

The calculated inductances are 6.0 × 10^−13^ H, 7.8 × 10^−13^ H, and 9.7 × 10^−13^ H, for Ring 1, Ring 2, and Ring 3, respectively.

The capacitance C can be obtained by:

8 $$C=\frac{Q}{{V}_{0}}=\frac{{\int }_{0}^{T/2}Idt}{{V}_{0}},$$

where T/2 is the half period of the incidence wave, and I is the surface current from the CST simulation. The calculated capacitances are 5.3 × 10^−17^ F, 5.0 × 10^−17^ F, and 5.2 × 10^−17^ F, for Ring 1, Ring 2, and Ring 3, respectively.

The current distributions are also fitted using Eq. (2) and plotted in Fig. 3 (solid curves) together with those from the CST simulation (dots, crosses, and diamonds). The comparison of the calculated R, V_0_, L and C values and those from the curve-fitting are listed in Table 1.

**Table 1:** Comparison of calculated and curve-fitting values.

	R (Ω)			V_0_ (10^−7^ V)			L (10^−13^ H)			C (10^−17^ F)		
Ring	1	2	3	1	2	3	1	2	3	1	2	3
Calculated	47.2	47.5	51.2	2.0	2.3	2.9	6.0	7.8	9.7	5.3	5.0	5.2
Curve-fitting	47.2	47.5	51.2	2.1	2.5	2.9	6.0	7.5	8.2	4.7	5.0	5.8

Note that in the calculation, the circular ring is treated as a perfect transmission line. No current distribution is considered across the width of the ring, whereas in the CST numerical simulation the current nonuniformity is counted. This causes the differences between the calcuted **R**, **V**_**0**_, **L** and **C** vlaues and those from the curve-fitting vlaues. As shown in Table 1, the values are quite close.

**Figure 4:**
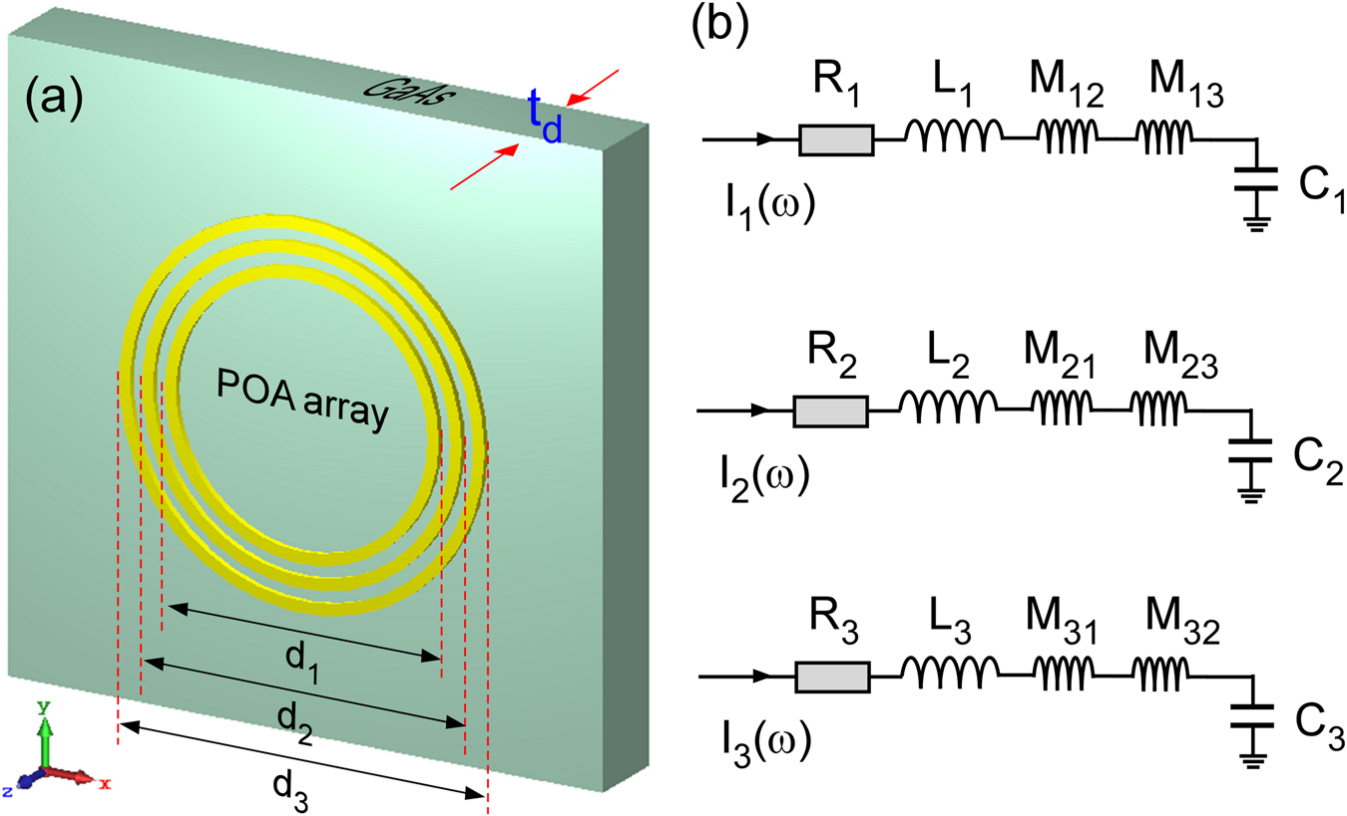
(**a**) layout of the concentric circular POA array; (**b**) Equivalent circuit for each ring. The extra inductors in each ring correspond to the couplings from the other rings.

After obtaining the parameters R, L, C for individual single rings, we then analyze the mutual couplings in a concentric circular ring POA array. Figure 4(a) shows the schematic layout of a three-ring POA array. The outer diameters of the rings are labeled as d_1_, d_2_, *et al*., and the widths of the rings are w_1_, w_2_, *et al*. Figure 4(b) shows the equivalent circuits for the rings of the POA array. The extra inductors in the equivalent circuit of each ring correspond to the mutual couplings from other rings.

In a matrix form the currents in coupled rings can be written as:

9 $$[\begin{array}{c}{I}_{1}\\ {I}_{2}\\ \ldots \\ {I}_{n}\end{array}]=[\begin{array}{cccc}\frac{1}{{Z}_{1}} & 0 & \ldots  & 0\\ 0 & \frac{1}{{Z}_{2}} & \ldots  & 0\\ \ldots  & \ldots  & \ldots  & \ldots \\ 0 & 0 & \ldots  & \frac{1}{{Z}_{n}}\end{array}]\,[\begin{array}{c}{V}_{10}\\ {V}_{20}\\ \ldots \\ {V}_{n0}\end{array}]+[\begin{array}{cccc}0 & \frac{j\omega {M}_{12}}{{Z}_{1}} & \ldots  & \frac{j\omega {M}_{1n}}{{Z}_{1}}\\ \frac{j\omega {M}_{21}}{{Z}_{2}} & 0 & \ldots  & \frac{j\omega {M}_{2n}}{{Z}_{2}}\\ \ldots  & \ldots  & \ldots  & \ldots \\ \frac{j\omega {M}_{n1}}{{Z}_{n}} & \frac{j\omega {M}_{n2}}{{Z}_{n}} & \ldots  & 0\end{array}]\,[\begin{array}{c}{I}_{1}\\ {I}_{2}\\ \ldots \\ {I}_{n}\end{array}],$$

10 $$[\begin{array}{c}{I}_{1}\\ {I}_{2}\\ \ldots \\ {I}_{n}\end{array}]={[\begin{array}{cccc}1 & \frac{-j\omega {M}_{12}}{{Z}_{1}} & \ldots  & \frac{-j\omega {M}_{1n}}{{Z}_{1}}\\ \frac{-j\omega {M}_{21}}{{Z}_{2}} & 1 & \ldots  & \frac{-j\omega {M}_{2n}}{{Z}_{2}}\\ \ldots  & \ldots  & \ldots  & \ldots \\ \frac{-j\omega {M}_{n1}}{{Z}_{n}} & \frac{-j\omega {M}_{n2}}{{Z}_{n}} & \ldots  & 1\end{array}]}^{-1}[\begin{array}{cccc}\frac{1}{{Z}_{1}} & 0 & \ldots  & 0\\ 0 & \frac{1}{{Z}_{2}} & \ldots  & 0\\ \ldots  & \ldots  & \ldots  & \ldots \\ 0 & 0 & \ldots  & \frac{1}{{Z}_{n}}\end{array}]\,[\begin{array}{c}{V}_{10}\\ {V}_{20}\\ \ldots \\ {V}_{n0}\end{array}],$$

11 $$[\begin{array}{c}{I}_{1}\\ {I}_{2}\\ \ldots \\ {I}_{n}\end{array}]={[\begin{array}{cccc}{Z}_{1} & -j\omega {M}_{12} & \ldots  & -j\omega {M}_{1n}\\ -j\omega {M}_{21} & {Z}_{2} & \ldots  & -j\omega {M}_{2n}\\ \ldots  & \ldots  & \ldots  & \ldots \\ -j\omega {M}_{n1} & -j\omega {M}_{n2} & \ldots  & {Z}_{n}\end{array}]}^{-1}[\begin{array}{c}{V}_{10}\\ {V}_{20}\\ \ldots \\ {V}_{n0}\end{array}],$$

where ${Z}_{n}={R}_{n}+j\omega {L}_{n}+\frac{1}{j\omega {C}_{n}}$ is the total impedance of an individual uncoupled ring.

Two different models are investigated to calculate the mutual induces. The first one is the mutual inductance of circular coils^31^ using Nagaoka’s formula^32^:

12 $$M={\mu }_{0}\sqrt{Aa}[4\pi {q}^{3/2}(1+\varepsilon )],$$

where A and a are the radii of the two circular coils and q and ε are the geometric parameters of the circular coils.

13 $$q=\frac{l}{2}+{(\frac{l}{2})}^{5}+{(\frac{l}{2})}^{15}+\ldots ,$$

14 $$l=\frac{1-\sqrt{k^{\prime} }}{1-\sqrt{k^{\prime} }},$$

15 $$k^{\prime} =\frac{\sqrt{{(A-a)}^{2}+{d}^{2}}}{\sqrt{{(A+a)}^{2}+{d}^{2}}},$$

where d is the distance between the two circular coils, and *d* = 0 for the concentric circular rings on the same plane.

16 $$\varepsilon =2{q}^{4}-4{q}^{6}+9{q}^{8}-12{q}^{10}\ldots ,$$

The mutual inductances calculated using the Nagaoka’s formula^32^ do not agree well with the simulation results. This is possibly due to the plane-wave induced non-uniform current distributions.

The second method is the mutual inductances of two parallel wires given by Rosa^33^:

17 $$M=\frac{{\mu }_{0}}{4\pi }(2{l}_{eff})[\mathrm{ln}(\frac{2{l}_{eff}}{s})-1+\frac{s}{{l}_{eff}}],$$

where *µ*_0_ is the permittivity of vacuum, *l*_*eff*_ is the effective length of a wire, and *s* is the separation of the two wires.

The *l*_*eff*_ can be written as:

18 $${l}_{eff}=\frac{\pi }{2}\frac{{R}_{out}}{\sqrt{2}},$$

where the factor 2 in the denominator is for the half-circle due to the plane-wave incidence induced symmetry, and the factor $\sqrt{2}$ accounts for the sinusoidal current distribution.

**Figure 5:**
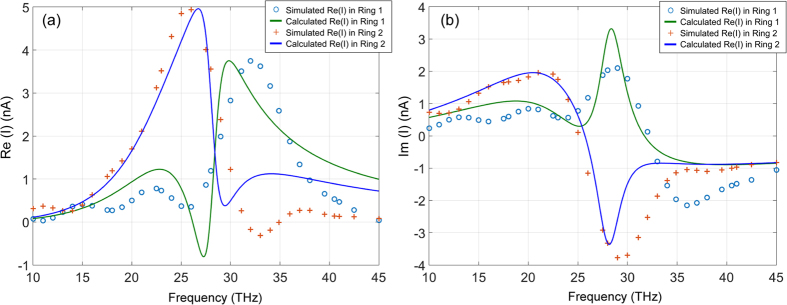
Comparison of the simulated and calculated currents in a two coupled ring POA array: (**a**) *Re*(*I*); (**b**) Im(I). The solid curves are the calculated currents using Eq. (11). The points by the “o” and “+” are numerical simulation data points using CST’s Microwave Studio®.

Figures 5(a) and 5(b) show the simulated (points) real *Re*(*I*) and imaginary *Im*(*I*) parts of the surface currents *I*_*m*_(*ω*) of two coupled circular rings compared with the calculated values using Eq. (5) (solid curves). The outer diameters of Ring 1, and Ring 2 are, 1.2 µm, 1.4 µm, respectively. The widths of the rings and thickness of the substrate are kept the same.

Table 2 lists the calculated mutual inductances *M*_12_ of different coupled rings using Eq. (17) compared with the mutual inductances from the curve-fitting.

**Table 2:** Mutual inductances *M*_12_ of different coupled rings.

Coupled rings	A	B	C
			
*M*_12_ (Calculated)	2.13 × 10^−13^ H	2.24 × 10^−13^ H	2.45 × 10^−13^ H
*M*_12_ (Curve-fitting)	2.07 × 10^−13^ H	2.22 × 10^−13^ H	2.47 × 10^−13^ H

The results calculated from the parallel wire model using Eq. (17) agree well with the curve-fitting. This indicates that under a plane wave illumination, the current distribution in a ring is similar to that in a wire transmission line.

**Figure 6:**
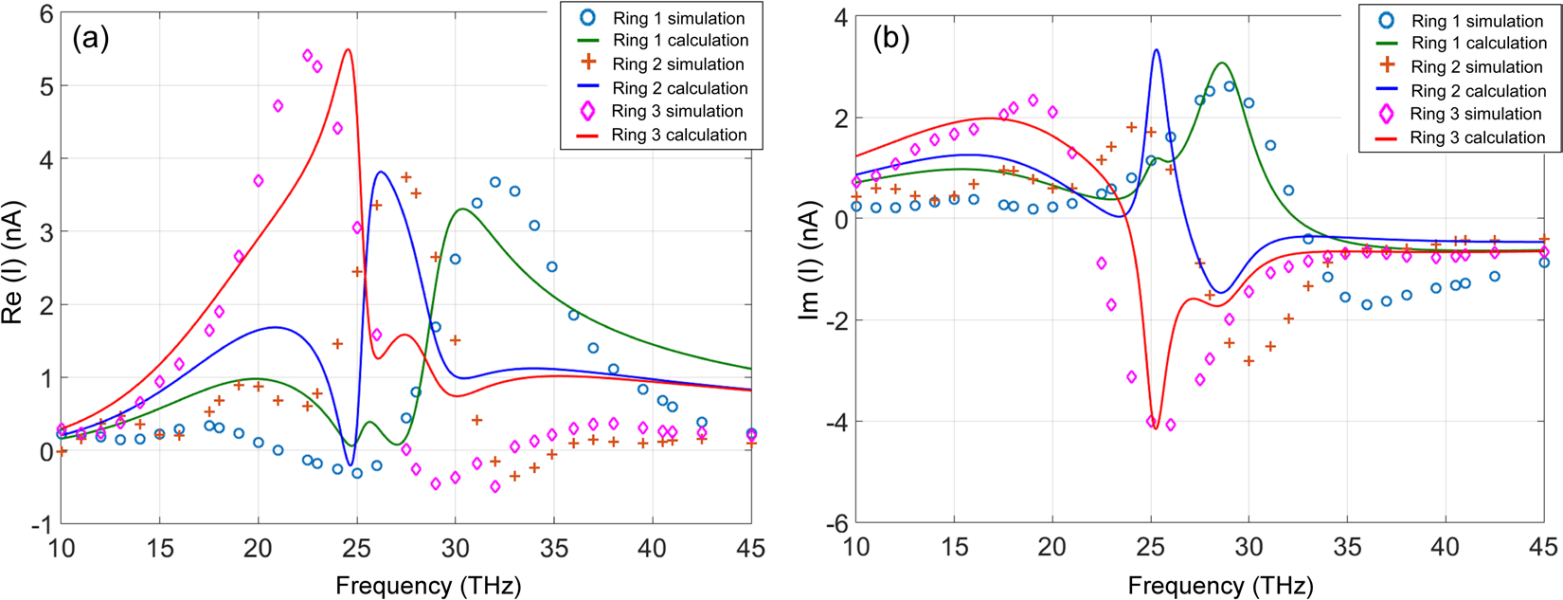
Comparison of the simulated and calculated currents in a three-ring POA array: (**a**) *Re*(*I*); (**b**) Im(I). The solid curves are the calculated currents using Eq. (11). The points by the “o”, “+” and “◊” are numerical simulation data points using CST’s Microwave Studio®.

Figures 6(a) and 6(b) show the calculated real and imaginary parts of currents (solid curves) in a three-ring POA array from Eqs (10) and (11) compared with the numerical simulation (circles, crosses, and diamonds). Table 3 lists the calculated mutual inductances using Eq. (17) compared with the values from the curve-fitting.

**Table 3:** Mutual inductances of different coupled rings.

Mutual inductances	*M*_12_	*M*_23_	*M*_13_
Calculated	2.13 × 10^−13^ H	2.91 × 10^−13^ H	2.34×10^−13^ H
Curve-fitting	2.07 × 10^−13^ H	2.79 × 10^−13^ H	2.28×10^−13^ H

The calculations agree well with the numerical simulation. The mutual couplings from the 2^nd^ nearest neighbors (i.e. *M*_13_, *M*_31_) are on the same orders as the nearest couplings.

## Conclusion

In conclusion, we develop an LCC model for the analysis of the mutual coupling in a concentric circular ring POA array. The current distributions in the circular rings and their mutual couplings are analyzed using the LCC model. The analytical calculations agree well the numerical simulation. The LCC model reveals the underlying mutual couplings between the rings in the POA array. It is found that the mutual couplings from the 2^nd^ nearest neighbors are not negligible. The LCC model provides a useful tool for the analysis of the near-field and their couplings in the circular ring POA array.
